# Arm race among closely-related carbapenem-resistant *Klebsiella pneumoniae* clones

**DOI:** 10.1038/s43705-022-00163-y

**Published:** 2022-08-22

**Authors:** Ying Liu, Shichao Zhu, Li Wei, Yu Feng, Lin Cai, Steven Dunn, Alan McNally, Zhiyong Zong

**Affiliations:** 1 Center of Infectious Diseases, West China Hospital, Sichuan University, Chengdu, China; 2 Center for Pathogen Research, West China Hospital, Sichuan University, Chengdu, China; 3 Department of Infection Control, West China Hospital, Sichuan University, Chengdu, China; 4 Intensive Care Unit, West China Hospital, Sichuan University, Chengdu, China; 5 Institute of Microbiology and Infection, College of Medical and Dental Science, University of Birmingham, Birmingham, UK

**Keywords:** Bacterial genomics, Bacterial infection, Antibiotics

## Abstract

Multiple carbapenem-resistant *Klebsiella pneumoniae* (CRKP) clones typically co-exist in hospital wards, but often certain clones will dominate. The factors driving this dominance are largely unclear. This study began from a genomic epidemiology analysis and followed by multiple approaches to identify the potential mechanisms driving the successful spread of a dominant clone. 638 patients in a 50-bed ICU were screened. 171 (26.8%) and 21 had CRKP from swabs and clinical specimens, respectively. Many (39.8% of those with ≥7-day ICU stay) acquired CRKP. After removing 18 unable to recover, 174 CRKP isolates were genome sequenced and belonged to six sequence types, with ST11 being the most prevalent (*n* = 154, 88.5%) and most (*n* = 169, 97.1%) carrying *bla*_KPC-2_. The 154 ST11 isolates belonged to 7 clones, with one (clone 1, KL64 capsular type) being dominant (*n* = 130, 84.4%). Clone 1 and the second-most common clone (clone 2, KL64, *n* = 15, 9.7%) emerged simultaneously, which was also detected by genome-based dating. Clone 1 exhibited decreased biofilm formation, shorter environment survival, and attenuated virulence. In murine gut, clone 1 outcompeted clone 2. Transcriptomic analysis showed significant upregulation of the ethanolamine operon in clone 1 when competing with clone 2. Clone 1 exhibited increased utilization of ethanolamine as a nitrogen source. This highlights that reduced virulence and enhanced ability to utilize ethanolamine may promote the success of nosocomial multidrug-resistant clones.

## Introduction

*Klebsiella pneumoniae* is one of the most common pathogens causing human infections. Carbapenems have long been mainstream antimicrobial agents against severe infections caused by *K. pneumoniae*. However, carbapenem-resistant *K. pneumoniae* (CRKP) has emerged as a major global challenge for clinical management and public health [1]. The production of carbapenem-hydrolyzing enzymes (carbapenemases) is the major mechanism mediating resistance to carbapenems in *K. pneumoniae*. There are a number of carbapenemase types and in *K. pneumoniae* the most common carbapenemase is KPC (a group of serine β-lactamases), followed by NDM and IMP (both of which are metallo-β-lactamases). The global dissemination of CRKP is largely mediated by the high-risk clonal complex 258 (CC258), which comprises ST11, ST258 and other closely related sequence types (e.g., ST340 and ST512) [2, 3]. CRKP strains within the same ST can be further differentiated into phylogenetically distinct clones. Multiple CRKP clones typically co-exist in individual healthcare institutions and their wards such as intensive care units (ICU). Individual clones are often more commonly seen and more widely distributed in the same institution or ward than others [4–9]. It has been well recognized that increased use of medical devices, overuse of antimicrobial agents, and low compliance of infection control are the main factors facilitating the emergence and dissemination of carbapenem-resistant *Enterobacteriaceae* [10–12]. However, the genetic and microbiological factors that predispose a given clone to be more successful in a healthcare setting than other clones from the same genetic background (ST) are yet to be determined.

In this study, we actively screened patients in a 50-bed ICU at a university hospital for CRKP and collected CRKP clinical strains. We performed whole genome sequencing on all CRKP isolates and identified a high level of acquisition of CRKP by patients within the unit, which was dominated by a clone belonging to ST11. Through comparative genomics, as well as a set of *in vitro* and larvae-based experiments we found that the dominant clone has attenuated virulence, which may facilitate long-term carriage. We also performed *in vivo* competition experiments in mice, and comparative transcriptomic analysis of clones. From this we determined that the dominant clone has enhanced ability to utilize ethanolamine, facilitating their intestinal colonization. This comprehensive approach incorporating genomics, laboratory experiments and *in vivo* studies sheds light on the microbiological factors (in this case attenuated virulence and increased utilization of ethanolamine) which drive the success of a CRKP clone in a healthcare setting.

## Results

### Few patients carried CRKP on ICU admission, but many acquired CRKP in the ICU

**Fig. 1:**
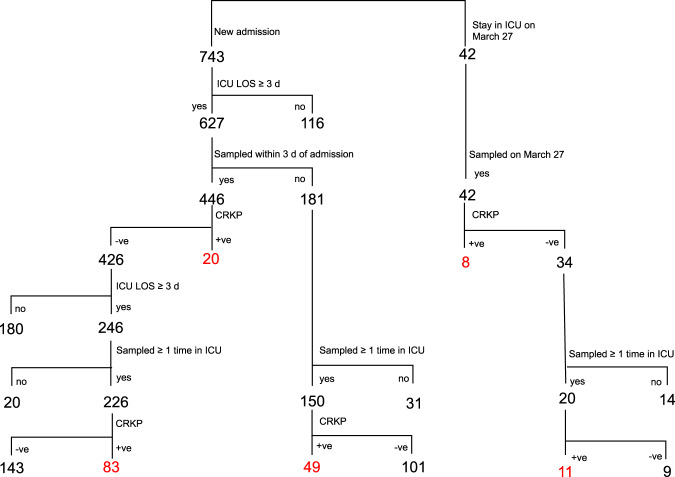
Schematic of patients swabbed in the study and those leading to isolation of CRKP. Patients staying within the ICU for more than 72 h were screened for CRKP using rectal swabs; where possible, swabs were collected within 72 h of their admission. For those patients who were CRKP negative on screening, and continued to stay for at least 7 days, we collected additional swabs to test for nosocomial acquisition. Patients sampled within 72 h of their admission showed a low colonization rate of just 4.5%. However, samples collected after 72 h, or indeed after patients had been within the ICU for some time, showed a much higher prevalence of CRKP (32.7–36.7%), indicating that CRKP is largely acquired within the ICU environment. Blue = patient cohorts, orange = sample collection, purple = CRKP negative, green = CRKP positive.

On March 27, 2017, there were 42 patients in the ICU who were screened via rectal swab, of which 8 (19.0%, 8/42) were found to harbour CRKP (Fig. 1). Of the 34 patients with no CRKP on admission, 20 stayed in the ICU for ≥7 days. Follow-up screening swabs were collected from all 20 patients, of which 11 had CRKP recovered from at least one rectal swab (Fig. 1).

During the study period, a total of 627 patients were admitted to the ICU and stayed within its care for 3 or more days. Rectal swabs were collected from 596 patients, with 74.8% (*n* = 446) of these swabs collected within 3 days of their admission, and 25.2% (*n* = 150) swabbed at some point afterwards. Amongst the 446 patients who were screened on admission, just 4.5% (*n* = 20) were found to harbour CRKP. For the 426 CRKP negative patients, 180 stayed within the ICU for less than 7 days and follow-up swab were not submitted and 246 stayed for at least 7 days, among which 226 actually submitted a follow-up swab, with CRKP subsequently recovered from 36.7% (*n* = 83) of samples (Fig. 1).

From the 150 primary samples that were collected after three days of ICU admission, CRKP was recovered from 32.7% (*n* = 49). The low CRKP carriage rate (4.5%) seen in those who were screened within 3 days of admission suggests that the vast majority (if not all) of the CRKP isolates in these 49 patients were acquired in the ICU. Therefore, a total of 638 patients were swabbed at least once, among which 171 had a CRKP isolate from rectal swabs during the study period (Fig. 1).

### Clinical impacts from CRKP are limited, but reflect CRKP carriage populations

Despite the relatively high carriage rates, very few patients developed clinical infections due to CRKP. Non-duplicate CRKP isolates were recovered from clinical samples of 21 patients in the ICU during the study period (Dataset S1 in the Supplementary file). From the 21 patients yielding clinical CRKP samples, 20 also had CRKP-positive rectal swabs; no rectal swabs were collected from the remaining patient. Among the 171 patients with CRKP colonization, 11.7% (20/171) developed infection due to CRKP.

### Most CRKP isolates belonged to ST11 and KL64 and carried *bla*_KPC-2_

Among the 171 CRKP isolated, 18 were non-culturable after storage. The remaining 153 CRKP isolates from rectal swabs and 21 from clinical samples (Dataset S1) were subjected to short-read whole genome sequencing. Therefore, a total of 174 CRKP isolates were sequenced.

The 174 CRKP isolates belonged to six STs, ST11, ST37, ST45, ST292, ST661, and ST1640 which is a single allele (*rpoB*) variant of ST11 (Supplementary Table S1 for the allele profile of the 6 STs). The vast majority (*n* = 154, 88.5%) of the isolates belong to ST11, with 16 isolates (9.2%) belonging to ST45, and a single isolate for the remaining four STs. In the ST11 isolates, 94.8% (*n* = 146) belonged to the KL64 capsular type, while the remaining 8 belonged to KL47 (*n* = 7) or KL39 (*n* = 1). All of the ST45 isolates belonged to KL62.

All CRKP isolates were resistant to carbapenems including meropenem (MIC, 4 to 512 mg/L) and imipenem (MIC, 4 to 512 mg/L) and were also resistant to aztreonam, ceftazidime, and piperacillin-tazobactam. Most of the isolates were also resistant to ciprofloxacin (resistance rate, 99.4%; 173/174), tigecycline (83.9% 146/174; MIC, 1 to 16 mg/L), amikacin (79.9%, 139/174), and trimethoprim-sulfamethoxazole (77.0%, 134/174). Only one isolate (carrying *bla*_NDM-1_, see below) was resistant to ceftazidime-avibactam (MIC, 512/4 mg/L). Similarly, only one isolate was resistant to colistin (MIC, 4 mg/L), and was found to carry a plasmid-borne colistin resistance gene, *mcr-1.1*. None of the isolates were resistant to aztreonam-avibactam.

Carbapenemase-encoding genes were found in all but two of the 174 CRKP isolates, with *bla*_KPC-2_ being detected in 169 isolates (97.1%), *bla*_KPC-12_ in two and *bla*_NDM-1_ in one. The two isolates with no known carbapenemase genes exhibited low-level resistance to carbapenems (MICs of imipenem and meropenem, 4 mg/L). Both isolates had a mutation or insertion in their OmpK35 and OmpK36 porins (details are provided in Supplementary Text S1), which have been found to result in reduced permeability of carbapenems [13], and have *bla*_CTX-M_ genes (*bla*_CTX-M-14_ and *bla*_CTX-M-15_ in one isolate and *bla*_CTX-M-3_ in another) encoding extended-spectrum β-lactamases.

### Co-occurrence of seven ST11 clones in the ICU with identification of a dominant clone 1 and a minor clone 2

**Fig. 2:**
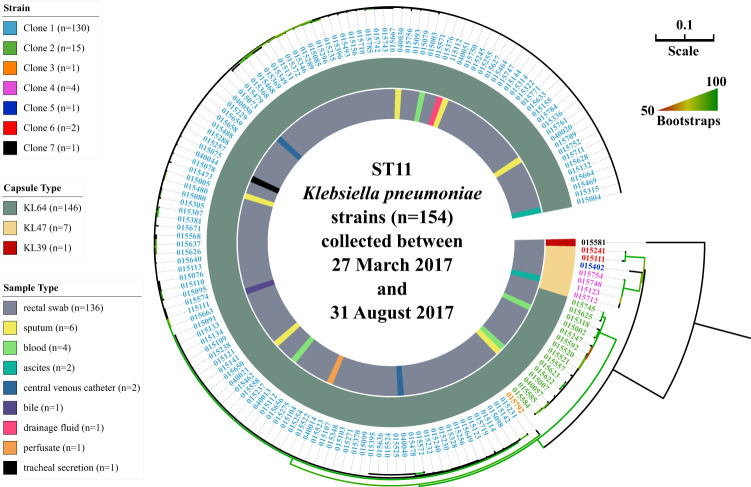
Phylogenomic tree of ST11 CRKP isolates (*n* = 154) collected in this study. The tree was inferred using strain 015093 as the reference. Information on the strains is available in Supplementary Dataset 1 and Dataset 2. The numbers of SNPs are shown in Supplementary Dataset 3. The phylogeny was inferred from core SNP sites under GTR model with site rate variation and a 1,000-bootstrap test. The tree was midpoint-rooted with bootstrap support over 50% shown in gradients. Strains from 7 well-separated clones were labelled with distinct colors which were clarified in the top-left corner.

We further typed ST11 isolates (*n* = 154) based on high-quality core SNPs. Strain 015093, the first isolate from a single patient who stayed in the ICU, was selected for long-read sequencing, allowing assembly into a complete genome (Supplementary Table S2). This complete genome of 015093 was used as the reference for phylogenetic analysis, with a total of 399 recombination-free variant sites shared by all isolates used to infer a phylogeny (Fig. 2). The 154 isolates could be robustly differentiated into seven distinct clones fully supported with 100% bootstraps, represented by clades (with multiple isolates) or branches (with a single isolate). Among these clones, those with the largest (*n* = 130) and the second largest (*n* = 15) populations, here designated as clone 1 and 2, belonged to the ST11 KL64 type (Fig. 2). The mean and the maximum intra-pairwise SNP distance of clone 1 and 2 were 6 and 9, and 18 and 20, SNPs, respectively, while the inter-pairwise SNP distance between these two ranged from 50 to 65 (Dataset S2 in the Supplementary). The remaining strains (*n* = 9) were located in two clades and three individual branches, here designated as clone 3 to 7. All were separated from each other by at least 35 SNPs on average (Dataset S2). The abovementioned seven clones could be assigned using a cutoff of 20 SNPs. Of the 21 clinically derived isolates, 67% (*n* = 14) were identified as clone 1, and 14% (*n* = 3) were identified as clone 2. This is largely reflective of the abundances in carriage (as determined by rectal swab screening), where 75.8% (*n* = 116) of the 153 rectal swab samples were assigned to clone 1, and 7.8% (*n* = 12) to clone 2 (Dataset S1).

### Clone 1 was introduced into the hospital after clone 2

**Fig. 3:**
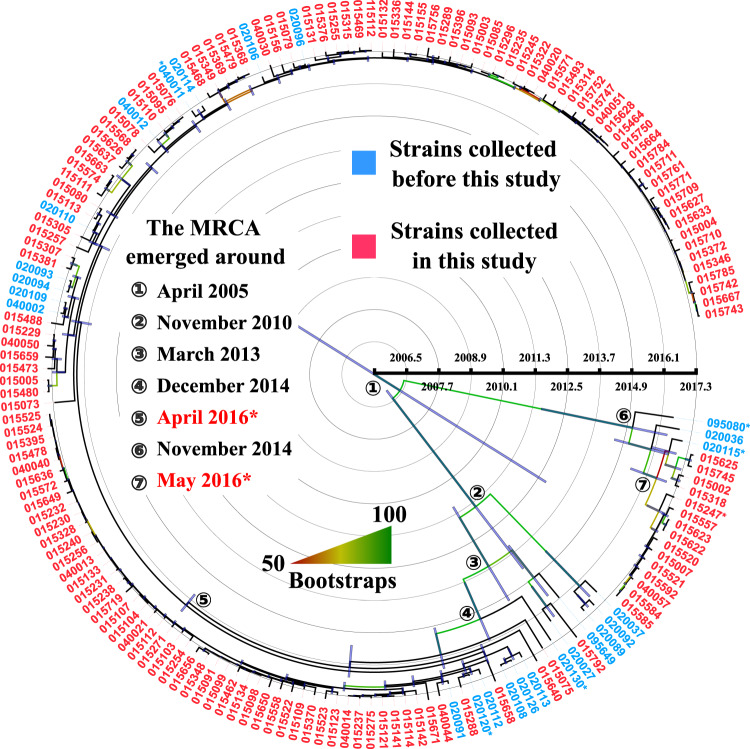
The time-calibrated phylogenetic tree of 171 strains of ST11 KL64 CRKP isolates in this study and collected in the hospital before the study. The dating was performed based on the maximum-likelihood tree inferred from core SNP sites under GTR model with site rate variation and a 1,000-bootstrap test. Strains collected in and before this study were highlighted in red and blue, respectively. Branches with bootstrap support over 50% were coloured in gradient and the average nucleotide substitution rate was estimated to be 4.85 per genome per year. MRCA refers to the most recent common ancestor. The 95% confidence intervals of estimated dates are as below: 1. October 1991 to May 2012; 2. November 2004 to February 2014; 3. May 2010 to November 2014; 4. May 2013 and December 2015; 5. September 2015 and July 2016; 6. January 2011 and April 2016; and 7. April 2015 and October 2016 with numbers 1 to 7 in circle. The estimated emergence of clone 1 and clone 2 corresponds to 7 and 5 marked with an asterisk, respectively.

It is possible that the dominant clone 1 may have emerged in our hospital prior to the initiation of this study. Whole genome sequencing of CRKP isolates recovered from clinical samples has been performed in West China hospital since 2014. To investigate when clone 1 and clone 2 emerged in our hospital, we interrogated our genome collection and included all ST11 CRKP isolates from 2014 to March 26, 2017 and created an additional SNP-based phylogenetic analysis as described above. There were 25 ST11 KL64 CRKP clinical isolates including 6 from the ICU between December 11, 2015 and March 26, 2017 (Dataset S3 in the Supplementary). Among these 25 strains, 16 belonged to clone 1, while 3 belonged to clone 2 (Fig. 3). Strain 020130, which was recovered from a non-ICU patient, was the closest strain to clone 1. Although there were only 9 to 18 core-genome SNPs between 020130 and clone 1 genomes, strain 020130 was not considered to be part of clone 1, as it formed a separate distinct branch in the phylogenomic tree (Fig. 3). Strain 020120 that was recovered on January 5, 2017 from a patient in the Emergency Department in this hospital was the earliest isolate of clone 1. The earliest isolate of clone 2 was strain 095080 recovered on June 18, 2016 from another patient, also in the Emergency Department. Both strain 020120 and strain 095080 were most likely introduced into the hospital by these patients as they were recovered from samples collected on the day of admission to the Emergency Department. The first isolate (strain 040011) of clone 1 in the ICU was recovered from a urine sample collected on March 16, 2017, while the first isolate (strain 015247) of clone 2 emerged in the ICU on May 9, 2019. Both 040011 and 015247 were likely introduced to the ICU as they were recovered from samples collected within 48 h of admission to ICU. Therefore, clone 1 was introduced into our hospital later than clone 2 but emerged in the ICU earlier than clone 2.

To estimate the date of the emergence of ST11 KL64 clones, we also inferred a time-calibrated phylogenetic tree (Fig. 3) based on the 171 strains with the effective sample size of all estimates over 400 to ensure the duration of MCMC chain was long enough. Using a relaxed clock model, the mean substitution rate was estimated 4.85 (3.31 to 6.68) substitutions per genome per year. The estimated emergence of clone 1 and clone 2 was dated April 2016 (95% CI, September 2015 to July 2016) and May 2016 (95% CI, April 2015 to October 2016), respectively (Fig. 3), suggesting that the two clones emerged almost at the same time. Despite this, clone 1 went on to colonize 8.6-fold more patients than clone 2, while circulating within the ICU over the same time-period.

### Clone 1 has decreased ability to form biofilms, shorter environmental survival, and attenuated virulence *in vitro*

Clone 1 has 13 unique SNPs including 11 in protein-coding sequences (CDS) and 2 in a spacer region (Supplementary Table S3), a truncated *ulaB* (Supplementary Fig. S1), and a 10.5-kb deletion which results in loss off type 1 fimbriae encoding *fim* genes (Supplementary Fig. S2, Table S4, and Table S5), when compared to clone 2 (Supplementary Text S2 for details). Only six SNPs were non-synonymous mutations. One of the missense mutations occurred in *rcsC*, which encodes a sensor kinase as part of the Rcs phosphorelay. The Rcs phosphorelay is conserved throughout the *Enterobacteriaceae* as an important signaling pathway and has also been found to actively participate in biofilm formation [14] and survival outside of the host [15]. Mutations of *rcsC* have been associated with decreased biofilm formation [16] and influence bacterial survival in environment [17]. In addition, there was a three-nucleotide insertion in *igaA*, leading to an aa insertion at position 662. *igaA* has been found to negatively regulate the Rcs phosphorelay [18–20].

**Fig. 4:**
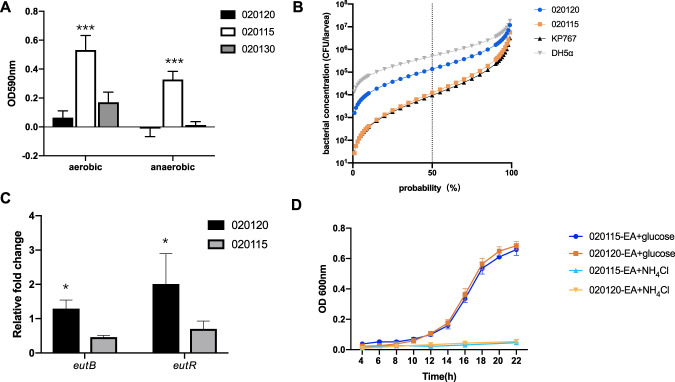
Biofilm formation, virulence assays, qRT-PCR, and ethanolamine utilization results. **A** Biofilm formation of C1_020120, C2_020115, and 020130. Absorption values of strains C1_020120, C2_020115, and 020130 (the closest strain of clone 1) at both OD _590 nm_ are shown. **B** Survival of *G. mellonella* after infection by bacterial strains. The effect of 1 × 10^3^, 1 × 10^4^, 1 × 10^5^, and 1 × 10^6^ CFU of each strain on survival of *G. mellonella* at 72 h after infection is shown. KP767, a hypervirulent *K. pneumoniae*, was used as a positive control, while *E. coli* DH5α was used as a negative control. **C** qRT-PCR for *eutB* (encoding the ethanolamine ammonia-lyase heavy chain) and *eutR* (encoding an HTH-type transcriptional regulator) genes for strain C1_020120 and C2_020115. Values are the mean ± standard deviation (SD). *p < 0.05. **D** utilization of ethanolamine (EA) as a sole carbon (adding NH_4_Cl) or nitrogen (adding glucose) source. C1_020120 and C2_020115 were able to use ethanolamine as a nitrogen source but not a carbon source.

Under anaerobic conditions, strain 020120 (the representative strain of clone 1, referred as C1_020120 thereafter to increase clarity) and strain 020130 (the closest strain of clone 1) exhibited no obvious biofilm formation (absorption at OD_590 nm_, mean ± standard deviations (SD), −0.01 ± 0.06 and 0.01 ± 0.02, respectively), while strain 020115 (the representative strain of clone 2, referred as C2_0201115 thereafter) formed biofilms (0.33 ± 0.06) (Fig. 4A). Under aerobic conditions, strain C1_020120 exhibited significantly less biofilm formation (0.06 ± 0.05) than strain 020130 (0.17 ± 0.07, *P* < 0.001) and strain C2_020115 (0.53 ± 0.10, *P* < 0.001) (Fig. 4A). In *in vitro* survival assays mimicking the ICU environment, *E. coli* ATCC 25922 survived only one day, while strain C1_020120 and C2_020115 survived 7 and 11 days (Supplementary Fig. S3), respectively.

The 50% lethal dose (LD_50_) at 72 h of strain C1_020120 against *G. mellonella* larvae was 1.4 × 10^5^ CFU, one log higher than the 1.2 × 10^4^ CFU of C2_020115, and higher than the 9.5 × 10^3^ CFU of the hypervirulent *K. pneumoniae* strain KP767 (Fig. 4B). Therefore, strain C1_020120 displays decreased virulence compared to the clone 2 strain.

### Clone 1 significantly outcompetes clone 2 in a murine intestinal colonization model

To confirm that mice had no CRKP intestinal carriage, a control group was orally administered meropenem for 3 days. No colonies were observed after overnight culture of fecal suspensions on SCAI medium containing 2 mg/L meropenem and 32 mg/L linezolid. Both tetracycline resistance in C2_020115 and amikacin resistance in C1_020120 were not transferrable and could therefore be used as the markers to differentiate the two strains.

**Fig. 5:**
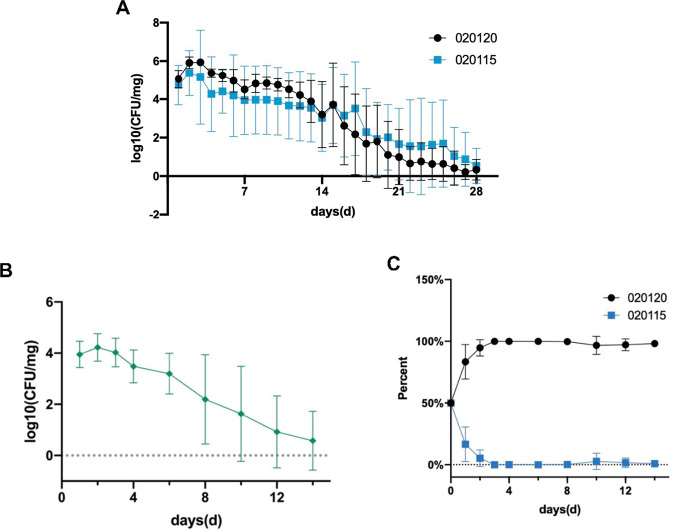
Murine intestinal colonization by C1_020120, C2_020115, and two strains in combination. **A** bacterial densities and the colonization days of fecal samples of mice fed with C1_020120 or C2_020115 alone. **B** bacterial densities and the colonization days of fecal samples of mice co-fed with C1_020120 and C2_020115 in combination. **C** the proportion of C1_020120 and C2_020115 in fecal samples of mice co-fed with C1_020120 and C2_020115 in combination.

In mice fed with either C1_020120 or C2_020115, the levels of colonization by both strains increased until day 2, and then steadily decreased for up to 28 days, with no significant level of difference between the two clones (Fig. 5A). A similar trend of colonization occurred in mice co-fed with both C1_020120 and C2_020115 (Fig. 5B). However, C1_020120 rapidly reach the highest density and then remained for up to 12 days. In contrast, levels of C2_020115 were almost undetectable by day three (Fig. 5C). This clearly shows that clone 1 has a significant fitness advantage over clone 2 when in direct competition in the intestines of mice.

### Ethanolamine and 1,2-propanediol metabolism pathways are significantly upregulated in clone 1 during competition with clone 2

**Fig. 6:**
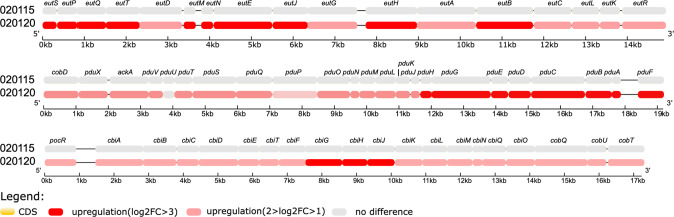
Differential expression level of C1_020120 or C2_020115 in ethanolamine, 1,2-propanediol, and cobalamin pathways. Differential expression level of C1_020120 or C2_020115 between when co-fed together and when fed alone in pathways for ethanolamine utilization (the *eut* gene cluster), 1,2-propanediol metabolism (the *pdu* gene cluster), and cobalamin biosynthesis (the *cbi/cob* gene cluster) are demonstrated in colours. The exact change in log2fold of these genes are shown in Dataset S5, and their function is listed in Dataset S4.

As C1_020120 and C2_020115 have highly similar genome sequences, we were unable to differentiate them in direct transcriptomic sequencing mice fecal samples co-colonizing both strains. As an alternative approach, we had to plate the mice fecal samples co-colonizing both strains in selective media to obtain the two strains separately, which were then subjected to transcriptomic sequencing. Although this approach would introduce confounding factors, we compared the separated *in vitro* growth of C1_020120 or C2_020115 on distinct selective media after passage together in murine gut to that of the same strain after passage alone, which also underwent *in vitro* growth on the same selective media to minimize confounding effects. Differential gene expression analysis of transcriptomic sequencing data revealed 74 significantly (>3 log2fold) upregulated genes in separated *in vitro* growth of C1_020120 on distinct selective media after passage together with C2_020115 in murine gut than that after passage alone (without C2_020115) (Dataset S4 in the Supplementary). In contrast, there were no significantly upregulated genes in C2_020115 in separated *in vitro* growth on distinct selective media after passage together with C2_020120 in murine gut. Among the 74 significantly upregulated genes, 50 have a functional annotation. Most of these (39/50) are involved in metabolic processes including propanediol utilization, ethanolamine utilization, and cobalamin biosynthesis. Interestingly, cobalamin (vitamin B12) is an essential part of the activation process for both ethanolamine and propanediol catabolism [21]. The ethanolamine utilization operon (the *eut* gene cluster), 1,2-propanediol operon (the *pdu* gene cluster), and the cobalamin biosynthesis operon (the *cbi* gene cluster) were significantly (>3 log2fold) upregulated in C1_020120 (Fig. 6, and Dataset S5 in the Supplementary). In contrast, none of ethanolamine and 1,2-propanediol metabolism pathways nor the cobalamin biosynthesis pathway had a significant change in levels of expression in C2_020115.

To confirm this finding, we examined the expression of *eutB* and *eutR* for C1_020120 and C2_020115 after passage in murine gut by qRT-PCR. We found that the expression of *eutB* and *eutR* of C1_020120 was significantly increased (1.30 ± 0.25 [*P* = 0.037] and 2.01 ± 0.88 [*P* = 0.046] folds, respectively) in separated *in vitro* growth on distinct selective media after passage together with C2_020115 in murine gut than that after passage alone. In contrast, the expression of *eutB* and *eutR* of C2_020115 in separated *in vitro* growth on distinct selective media after passage together with C1_020120 was only 0.46 ± 0.05 and 0.71 ± 0.23 folds, respectively, of that after passage alone (Fig. 4C). On the other hand, we also compared the expression of *eutB* and *eutR* between C1_020120 and C2_020115 in separated culture in LB broth and after separate passage in murine gut. We did not find significant differences in the expression of *eutB* and *eutR* between C1_020120 and C2_020115 in broth culture (*P* = 0.372 and *P* = 0.368) nor after passage alone in murine gut (*P* = 0.492 and *P* = 0.481) (Supplementary Fig. S4).

### Clone 1 has a fitness advantage over clone 2 in the presence of ethanolamine as a sole nitrogen source

Previous studies have found that ethanolamine can be used as nitrogen and carbon sources by some bacteria [22, 23]. We found that both C1_020120 and C2_020115 were able to use ethanolamine as a nitrogen source by measuring OD_600 nm_ of individual overnight bacterial cultures (mean ± SD, 0.68 ± 0.03 and 0.66 ± 0.04, respectively), but not a carbon source (0.05 ± 0.01 and 0.05 ± 0.00, respectively) (Fig. 4D). When both strains were co-cultured in the presence of ethanolamine as the sole nitrogen source, C1_020120 had a clear fitness advantage (w, 1.19 ± 0.15) over C2_020115.

### A chromosomal inversion occurs within a prophage upstream of the *eut* operon in C1_020120

**Fig. 7:**
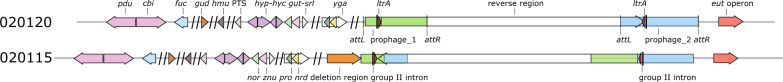
The reversion of a region between the *pdu*-*cbi* module and the *eut* operon. Compared to that of C2_020115, a large region between the module of *pdu* (for utilizing 1,2-propanediol) and *cbi* (for cobalamin biosynthesis) of C1_020120 was reversed. This reversed region is flanked by two identical 226-bp group II introns and their reverse transcriptase (RT)-encoding genes *ltrA*. In strain C1_020120, the two group II introns and *ltrA* genes belong to two different prophages, 61.3-kb prophage_1 (in light green) and 53.9-kb prophage_2 (in cyan), respectively. The two prophages are intact and have the attachment sites, *attL* and *attR*, at both sides. Homologous recombination between the two group II introns could reverse the intervening region including parts of the two prophages. The 10.5-kb region absent from clone 1 (Fig. S2) is also located between the *pdu*-*cbi* module and the *eut* operon and is indicated as ‘deletion region’ here. Other genes shown are *fuc* (encoding metabolism for L-fucose), *gud* (encoding glucarate dehydratase), *hmu* (encoding an ABC transporter complex involved in hemin import), PTS (encoding aphosphoenolpyruvate-dependent sugar phosphotransferase system), *hyp-hyc* (involving in hydrogenase biosynthesis), *gut-srl* (involving in metabolism of the hexitol D-glucitol), *yga* (encoding inner membrane and ribosome binding proteins with putative rhodanese activity), *nor* (for the expression of anaerobic nitric oxide [NO] reductase), *znu* (involving in the high-affinity zinc uptake transport), *pro* (involving in glycine betaine and proline betaine uptake), and *nrd* (encoding a ribonucleotide reductase system). This figure is not scaled.

There are no SNPs or indels in clone 1 which would account for a such a significant phenotypic adaptation in comparison to clone 2. We, therefore, re-analyzed the combined long-read and short-read complete genome assemblies of C1_020120 and C2_020115 to look for structural rearrangements. We discovered that the 10.5-kb deletion resulting in loss off type1 fimbriae encoding *fim* genes (Fig. S2) in clone 1 occurred in the region of the genome containing the *pdu-cbi* and *eut* operons (Fig. 7). Furthermore, C1_020120 and C2_020115 had a rearrangement (reversion) of a large 160-kb region due to the recombination between the flanking two 226-bp group II introns, switching confirmation (Fig. 7). In strain C1_020120, The two group II introns and their reverse transcriptase-encoding genes *ltrA* belong to two different intact prophages, 61.3-kb and 53.9-kb in size, respectively. There were no other structural or genome architecture differences between the two clones, and no other SNPs or indels which would affect the expression of the *eut* operon. Mapping reads of all our ST11 isolates against the clone 1 reference genome confirmed that all clone 1 isolates have this structural rearrangement, which is notably absent from clone 2. This 160-kb region was also found to be fully conserved across all publicly available and fully completed *K. pneumoniae* genomes, which consisted of 205 STs (*n* = 1,012, accessed on 2021-10-28, Dataset S6). In the vast majority of genomes (*n* = 997, 98.5%), this region occurs in the same orientation as clone 1, with only 15 genomes matching the orientation found in clone 2.

## Discussion

*Klebsiella pneumoniae* is amongst the 12 priority pathogens that the World Health Organisation classified as of critical importance to global human health. While many studies have assessed the clinical burden of *K. pneumoniae*, here we also report alarming rates of CRKP carriage amongst patients within an ICU, highlighting an urgent problem for modern healthcare. Active screening of patients in an ICU revealed just 3.8% of CRKP carriage on ICU admission, but ultimately, 39.8% of patients who stayed in the ICU for at least 7 days acquired CRKP during their stay. This increasing prevalence of CRKP within a hospital environment is of great concern. Not only because a moderate number of patients go on to develop a disease directly mediated by CRKP (11.7%); but also because the *K. pneumoniae* isolates that are circulating within the hospital contain multiple antimicrobial resistance genes that can be exchanged via horizontal transfer.

The high acquisition rate of CRKP within the ICU was driven by a dominant clone of ST11 and KL64 capsular type, described here as clone 1. This clone emerged within the hospital at the same time as a much less successful clone (clone 2). Using genomic and transcriptomic analysis as well as laboratory and animal experiments, we show that attenuated virulence and the enhanced ability to utilize ethanolamine in the intestinal tract may contribute to the success and dominance of clone 1 in this nosocomial setting.

Attenuated virulence would clearly benefit the clone in being more successful at asymptomatic colonization of the intestinal tract. A suggestion borne out by the fact that only 11.7% of the patients screened during this study went on to exhibit a clinical infection from CRKP. Reduced virulence would likely incur a weaker inflammatory response from the host immune system, something that has been shown to occur in ST171 *Enterobacter xiangfangensis* of the *Enterobacter cloacae* complex, a globally-disseminated lineage of carbapenem-resistant *Enterobacter* [24]. The decrease in biofilm formation and survival in environmental conditions observed in clone 1 also points towards selective adaptation to gut colonization. This is a hypothesis that has been previously posited for other multi-drug resistant (MDR) clones such as the B4/H24RxC MDR clone of *E. coli* ST410, where a recombination in the *fhu* iron acquisition genes was the dominant genetic event in the formation of the MDR clone [25]. Our results also show that the prevalence of CRKP strains derived from clinical samples are reflective of the CRKP populations circulating within the ICU. It has been suggested that the ability of a particular strain to colonize the human intestinal tract may be a key driver of relative clinical success, rather than an inherently greater virulence or pathogenicity [26]. This further highlights the importance of screening studies to fully understand the epidemiology of *Klebsiella*, both as priority pathogens, and as reservoirs of antimicrobial resistance.

Indeed, there is a growing body of evidence that the formation of MDR clones is underpinned by selection in genes encoding metabolic processes [27] and other functions involved in mammalian colonization [28]. In this study, we show that enhanced ability to utilize ethanolamine may provide an advantage to clone 1 being dominant over other nosocomial circulating clones. Ethanolamine is a breakdown product of the membrane phospholipid phosphatidylethanolamine and is abundant in the intestinal tract [29–31]. Many bacteria including *Klebsiella* species have the ability to use ethanolamine as a sole source of nitrogen and sometimes carbon [22, 23]. Scavenging nutrients is a key factor for successful bacterial colonization in the host intestinal tract [23]. Previous studies have found that the ability to metabolize ethanolamine promotes growth of invasive bacterial species and therefore provides a competitive advantage for these strains to colonize the intestinal tract [23, 30, 32]. Ethanolamine may also serve as a signal that allows the bacterium to sense the intestinal environment and then trigger the appropriate gene expression for intestinal colonization. We detected a prophage mediated chromosomal inversion upstream of the *eut* operon in clone 1, and further characterized this inversion in the majority of complete *Klebsiella* genomes that were retrieved from NCBI. Given the largely clinical bias of existing *K. pneumonia* research, this may represent a mechanism under which ethanolamine metabolism is modulated in clinically successful clones. Alternatively, the elevated expression of the *eut* operon may not result from the inversion or it alone but could be due to other, yet-to-be-identified, factors or a combination of multiple factors comprising the inversion. This warrants further studies.

During co-colonization within a mouse model, we observed the up-regulation of the *eut* and *cbi* operons in the dominant clone 1, and simultaneous down-regulation in clone 2. Given the finite abundance of ethanolamine within the intestine, and the increased ability of clone 1 to utilize ethanolamine, this may represent a mechanistic basis for competition within the intestinal tract whereby clone 1 utilizes all available ethanolamine, clone 2 is simply not able to effectively compete for this nitrogen source, which ultimately leads to switching off of the ethanolamine and vitamin B12 dependent transcriptional cascade [33] in clone 2.

## Conclusion

Our study suggests that the selection observed for maintenance of the *pdu/cbi* and *eut* operons over long times scales in human pathogens [34] may also be an important selective force facilitating the spread of MDR clones in nosocomial settings. These findings could significantly impact our understanding of the evolution of dominant antimicrobial resistant clones and inspire new targeted studies to address the control of clinically significant MDR microorganisms at the level of intestinal colonization.

## Methods

### Study design

This study began as a genome sequence-based study aiming to investigate the clonal transmission of CRKP in a 50-bed general ICU at West China Hospital, which is a major referral medical center in western China, between March 27, 2017 (the date of the initiation of active screening for CRKP among patients hospitalized in the ICU) and August 31, 2017. The study was approved by the Ethical Committee of West China Hospital, Sichuan University, and informed consent was waived.

### Active screening of ICU patients for CRKP

Patients were screened within 3 days of admission to the ICU and thereafter on a weekly basis during their stay in the ICU. Rectal swabs were collected and were streaked onto Simmons Citrate Agar Inositol (SCAI) medium [35, 36] agar plates containing 2 mg/L meropenem, which were cultured for 2 days. Yellow colonies indicative of *K. pneumoniae* were picked [35], and their identification was confirmed using matrix-assisted laser desorption/ionization time-of-flight mass spectrometry (MALDI-TOF; Bruker, Billerica, MA, USA).

### Clinical isolates of CRKP

All non-duplicate CRKP isolates that were recovered from clinical samples of patients in the ICU between March 27, 2017 and August 31, 2017 were collected. Initial species identification was performed using Vitek II (bioMérieux, Marcy-l'Étoile, France). All CRKP isolates from clinical samples and rectal swabs were stored at −80 °C.

### *In vitro* susceptibility testing

Minimum inhibitory concentrations (MICs) of amikacin, aztreonam, aztreonam-avibactam, ceftazidime, ceftazidime-avibactam, ciprofloxacin, colistin, imipenem, meropenem, piperacillin-tazobactam, tigecycline, and trimethoprim-sulfamethoxazole were determined for all CRKP strains from clinical samples and rectal swabs using the broth microdilution method of the Clinical and Laboratory Standards Institute (CLSI) [37]. The breakpoints of aztreonam defined by CLSI were applied for aztreonam-avibactam. As there are no breakpoints of tigecycline from CLSI, those defined by EUCAST (http://www.eucast.org/) for *Escherichia coli* (susceptible, ≤0.5 mg/L; resistant, >0.5 mg/L) were applied.

### Short read-based genome sequencing and analysis

CRKP isolates from clinical samples and rectal swabs of each patient were subjected to whole genome sequencing. For patients with multiple CRKP isolates from rectal swabs or clinical samples, the first isolate was sequenced. For patients with multiple CRKP isolates from rectal swabs and clinical samples, both the first isolate from a swab, and the first isolate from a clinical sample were sequenced. Genomic DNA of all CRKP strains from clinical samples and rectal swabs was prepared using QIAamp DNA Blood Mini Kit (Qiagen; Hilden, Germany) followed by the library construction aiming for 350 bp in length using the NEBNext Ultra II DNA Library Prep Kit for Illumina (NEB; Ipswich, MA, USA). High-throughput-sequencing was performed on the Hiseq-10X platform (Illumina; San Diego, CA, USA) with a paired-end layout of 150 bp. The quality of paired-end short reads was initially controlled by sequentially removing 10 bp from each end, trimming low-quality base (<Q15) at the end and adaptor contamination using Cutadapt v2.10 [38] and BBTools v38.86 (https://jgi.doe.gov/data-and-tools/bbtools/) to obtain clean reads, which were then fed into the SPAdes-based assembly pipeline Shovill v1.1.0 (https://github.com/tseemann/shovill) with default settings.

### Strain typing and gene prediction

A quality check was performed on the assembled genomes using CheckM v1.0.18 [39] to determine the existence of contamination. Genomes passed this quality check were annotated using Prokka v1.14.5 [40]. The precise species identification was established based on average nucleotide identity (ANI) between the strains and type strains of *Klebsiella* species using JSpeciesWS [41]. The sequence type (ST) was determined using mlst v2.18.0 (https://github.com/tseemann/mlst) which queries the PubMLST database (https://pubmlst.org/). Capsule typing and virulence factors prediction and were performed using Kleborate v2.0.0 [42] and Kaptive [43]. Antimicrobial resistance genes were predicted using AMRFinderPlus v3.9 [44]. Plasmid replicons were identified using PlasmidFinder [45] and replicon sequence typing for IncFII plasmids was determined using pMLST (https://cge.cbs.dtu.dk/services/pMLST/) [46]. For isolates with no known carbapenemase genes, alterations of OmpK35 and OmpK36, the two major nonspecific porins of *K. pneumoniae*, were examined using Kleborate v2.0.0 [42]. Prophages were identified using PHASTER [47].

### Long read-based genome sequencing and analysis

The first strains (according to the sample collection date) of each of the two major clones belonging to ST11, C1_020120 (the representative strain of clone 1) and C2_020115 (the representative strain of clone 2, see below for details), and strain 015093, the first one isolated from a patient who stayed in ICU for the entire study were selected for the long-read MinION Sequencer (Nanopore; Oxford, UK). For those subjected to the long-read sequencing, their DNA was extracted using Monarch® Genomic DNA Purification Kit (NEB). The long-read library was prepared with multiplexing and sequenced according to the manufactures’ guide using flow cell R9.4.1 (Nanopore). The quality of long reads was controlled by being mapped with their corresponding short reads using Filtlong v0.2.0 (https://github.com/rrwick/Filtlong) with minimum quality and length as Q8 and 2,000 bp respectively. Along with the corresponding clean reads, long reads were fed into the hybrid assembler Unicycler v0.4.8 [48] and run under the conservative mode.

### Determining clonal relatedness by SNPs analysis

The chromosomal sequence of strain 015093 obtained from the hybrid assembly of MinION/Illumina sequencing reads was used as the reference for mapping. Quality-controlled short reads of each strain were mapped against the selected complete genome for identifying single nucleotide polymorphism (SNP) using pipeline Snippy v4.6.0 (https://github.com/tseemann/snippy) with default settings. A pseudo-alignment of chromosomes was generated by applying SNPs of each strain onto the reference genome, followed by recombination filtering using Gubbins v2.4.1 [49] with a maximum 50 iterations under the GTR-GAMMA model. The recombination-free maximum-likelihood tree was then inferred using RAxML v8.2.12 [50] under the GTR-GAMMA model with 1,000 iterations of bootstrapping. The distinction of ST11 lineages was based on the topological structure, branch support, and the significance of difference in SNP among intra- and inter-lineages. Briefly, topologies and bootstraps were challenged by re-inferring the phylogenetic trees using three more distantly related references, and statistical significance was assessed using two-tailed Student’s *t*-tests. By incorporating the collection date of individual strains, the maximum-likelihood tree was dated using BactDating v1.0.12 [51] under the mixed model with a 10^7^-iteration Markov Chain Monte Carlo (MCMC) chain. Trees were viewed and annotated using FigTree v1.4.4 (http://tree.bio.ed.ac.uk/software/figtree) and iTOL v5.7 [52].

### Dating the emergence of two most successfully spread ST11 clones

Bacterial clones may have emerged in our hospital prior to the initiation of this study. We have performed whole genome sequencing CRKP isolates recovered from clinical samples in our hospital since 2014. To investigate the time when the two most successfully spread clones (clone 1 and clone 2) emerged in our hospital, we retrospectively examined our strain collection and included all ST11 CRKP isolates (Dataset S3) from 2014 to March 26, 2017 (the start of this project) into an additional phylogenetic analysis based on SNPs as described above.

### Identifying loci specific to ST11 clone 1

Pan-genome matrixes were created using Roary v3.13.0 [53] with different identity criteria (85%, 90%, 95% or 98%) and without splitting paralogs. In conjunction with this, the intergenic regions (IGRs) were also compared using Piggy v1.5 [54]. A similar matrix of all SNP sites was created from the pseudo-genome alignment using SNP-sites v2.5.1 [55]. The association between traits and genes, traits and SNPs, and traits and IGRs were then calculated using Scoary v1.6.16 [56]. Gubbins v2.4.1 [49] was used to determine whether the clone-specific genes and SNPs were due to recombination. SNPs in the −10, −35 boxes of promoter or in the 5′ UTR regions of downstream genes were predicted using the online tool BPROM (http://www.softberry.com/) [57].

### Coalescent analysis and transmission route prediction

The pseudo-genome alignment of strains subjected to coalescent analysis was created and searched for recombination sites using Snippy and Gubbins as described above. These outputs along with the isolation dates in unit of years were fed into BactDating v1.0.12 [51] running under mixed model for 10^8^ iterations of MCMC or until the effective sample size of all inferred parameters α, μ and σ reached 200.

### Biofilm formation assays

Strain C1_020120 (representative strain of clone 1, see below) was subjected to a biofilm formation assay with strain 020130 (the closest strain of clone 1) and strain C2_020115 (representative strain of clone 2) used as controls under both aerobic and anaerobic conditions as described previously [58, 59]. Briefly, bacterial cells were harvested from overnight cultures in LB broth by centrifugation at 2,500 r/min for 10 min and resuspended with saline and were adjusted to 0.5 McFarland standard. Aliquots (100 μl) were then pipetted into 96-well polystyrene culture plates and incubated for 3 h at 37 °C in an incubator or in an anaerobic bag (bioMérieux) to allow the formation of biofilms. The plates were washed twice with distilled water. Biofilms in the wells were fixed with 100 μl formalin per well for 15 min and were stained with 100 μl staining buffer containing 1% crystal violet for 5 min. The stained biofilms were washed again to remove the unbound stain and allowed to dry at room temperature. Biofilms were detected with 100 μl 30% glacial acetic acid by ELX800 Universal Microplate Reader (Bio-Tek, Winooski, VT, USA) at OD_590 nm_. The biofilm formation assay was repeated a total of nine times for each strain.

### Survival assay in environments mimicking the ICU

*In vitro* survival of strains C1_020120 and C2_020115 was assayed on dry surfaces as described previously [60, 61] with *E. coli* ATCC 25922 as a control. Briefly, bacterial cells were harvested from overnight cultures in LB broth by centrifugation at 2,500 r/min for 10 min, resuspended with phosphate-buffered saline (PBS) and adjusted to a 0.5 McFarland standard. Aliquots (10 μl) were then pipetted into 96-well polystyrene culture plates with three wells per plate for each strain to repeat the assay in triplicate. Plates were dried at room temperature overnight and were then placed in a corner of the ICU to reflect the real temperature and humidity of the ICU. Every two days, a plate was retrieved, and each well was resuspended with 200 μl PBS. A 100 μl aliquot of each well was streaked onto a LB agar plate, which was incubated at 37 °C overnight. Survival days were determined using the last day of bacterial growth in at least two of the three replicates.

### *In vitro* virulence assay

The virulence of strains C1_020120 and C2_020115 was assessed using wax moth (*Galleria mellonella*) larvae weighing between 200 to 300 mg (Gaoge; Shanghai, China). A hypervirulent *K. pneumoniae* strain, KP767 [62], was used as a positive control, while *E. coli* DH5α was used as a negative control. Overnight bacterial cultures were washed using PBS and were further adjusted with PBS to concentrations of 1 × 10^5^, 1 × 10^6^, 1 × 10^7^, and 1 × 10^8^ CFU/ml. Larvae (*n* = 16) were injected with 10 μl of inoculum into hemocoel via the last left proleg using a 25-μl Hamilton syringe [63]. The infected larvae were then incubated in plastic containers at 37 ^o^C. The number of live larvae was counted every 12 h for 3 days. Assays were performed in triplicate. Probit analysis was used to determine the lethal doses at which 50% (LD_50_) of the infected larvae would be killed at 72 h following injection.

### Conjugation experiments

Strain C1_020120 was susceptible to tetracycline (MIC, 4 mg/L), while strain C2_020115 was resistant (MIC, >512 mg/L). Similarly, strain C1_020120 was resistant to amikacin (MIC, >512 mg/L), while strain C2_020115 was susceptible (MIC, 8 mg/L). Tetracycline and amikacin may be used as the selection marker to differentiate the two strains in head-to-head competitions. However, if the tetracycline resistance of strain C2_020115 or the amikacin resistance of strain C1_020120 could be transferred by conjugation, such differentiation using tetracycline or amikacin may be blurred. We, therefore, performed conjugation experiments in broth and on filters as described previously [64–66] to examine whether tetracycline and amikacin resistance was transferrable.

The azide-resistant *E. coli* strain J53 AizR and *K. pneumoniae* 700603 AziR (azide-resistant variant of J53 and ATCC 700603, respectively) were used as the recipient. For filter-based mating, overnight donor cultures (1 mL) were harvested by centrifugation, washed twice with 1 mL saline, and re-suspended in 100 µL saline. Recipient cells were harvested from plates using a bent Pasteur pipette, washed, and suspended in 500 µL saline. Donor and recipient suspensions were mixed (50 μL each). The mixture was placed on a 0.45 μM cellulose-ester filter (Xinya; Shanghai, China) and then incubated on a blood agar plate at 37 °C for 4 h. Subsequently, the mixture of cultures was harvested in 1 mL saline, centrifuged, and re-suspended in 200 μL saline. For broth-based mating, overnight cultures of donor (25 µL) and recipient strains (250 µL) were added to 3 mL fresh brain heart infusion (BHI) broth. The mixture was incubated for 18 h at 37 °C without shaking. Potential transconjugants were selected on LB agar plates containing 64 mg/L tetracycline and 150 mg/L sodium azide.

### *In vivo* competition assay using the murine gut colonization model

All animal experiments in this study were approved by the Ethics Committee for Laboratory Animals of West China Hospital, Sichuan University, Chengdu, China. We used C57BL/6J mice that were housed with good care in the Laboratory Animal Centre at Sichuan University for *in vivo* competition experiments. Twenty-eight seven-week-old female mice weighted 18 to 20 g were randomly assigned into four groups including one control and three experimental groups. After being fed normally for seven days, mice of all groups were orally administered 50 mg/L meropenem for three days. C1_020120 and C2_020115 were grown separately in 5 ml LB broth overnight at 37 °C. The cultures were harvested by centrifuging and the generated bacterial pellets were washed by sterile PBS, resuspended in 1 ml sterile PBS, measured to the 0.5 McFarland standard, and finally adjusted to 5 × 10^4^ CFU/ml. Mice in the three experimental groups were then mouth-fed with 200 μl bacterial suspensions containing about 10^4^ cells of C2_020115, C1_020120, or the mixture of the two isolates at a 1:1 ratio, respectively. For control, mice were mouth-fed with 200 μl sterile PBS. Fecal pellets were collected from individual mice on the subsequent day after inoculation and then every day and were streaked onto SCAI medium [35, 36] containing differential antimicrobial agents to enumerate bacterial cells. Plates containing 2 mg/L meropenem, 32 mg/L linezolid and 50 mg/L amikacin were used for screening C1_020120, while plates containing 2 mg/L meropenem, 32 mg/L linezolid, and 64 mg/L tetracycline were used for screening C2_020115. Linezolid was added to inhibit the growth of Gram-positive bacteria such as *Enterococcus* spp. The fecal samples were stored at −80 °C in a resuspension supplemented with 1 ml glycerol described.

### Transcriptome sequencing and analysis

Strains C1_020120 and C2_020115 were used as the representative strain of clone 1 and clone 2, respectively. Fresh feces collected from mice of the three groups (orally fed by C1_020120 alone, C2_020115 alone, and C1_020120 and C2_020115 in combination) were separately weighted and suspended in 1 ml PBS. A 100 μl aliquot from each of three groups of suspensions were streaked onto a SCAI agar plate containing 2 mg/L meropenem, 32 mg/L linezolid, and 64 mg/L amikacin (for selecting C1_020120) or 64 mg/L tetracycline (for selecting C2_020115). After incubation at 37 °C overnight, bacterial cells were harvested from each agar plate using sterile inoculation loops and were suspended with 1 ml PBS. Three biological replicates of each group were obtained using the above procedure for further studies. All samples were confirmed to be free of contamination by the other strain by PCR of *rmtB* (to detect C1_020120) or *tetA* (to detect C2_020115). All samples were frozen in liquid nitrogen immediately, stored at −80 °C and used in subsequent experiments within 5 days.

RNA was isolated using the RNAprep Pure Cell/Bacteria Kit (TIANGEN BIOTECH; Beijing, China). Purity and integrity of RNA were assessed using 1% agarose gels and the RNA Nano 6000 Assay Kit of the Bioanalyzer 2100 system (Agilent Technologies; Santa Clara, CA, USA). Library fragments were constructed using the NEBNext Ultra Directional RNA Library Prep Kit for Illumina (NEB). After cluster generation of index-coded samples using TruSeq PE Cluster Kit v3-cBot-HS (Illumina), the library preparations were sequenced on an Illumina Novaseq platform with a 150 bp paired-end protocol by Novogene Inc. (Beijing, China).

Clean data were obtained by read filtering using fastp from raw data. The complete genome sequences of C1_020120 and C2_020115 (see above) were used as the reference. Clean reads were aligned with reference genomes using Bowtie2 [67] and the numbers of reads mapped to each gene were counted using HTSeq v0.6.1 [68]. Differential gene expression analysis of two groups (the strain [either C1_020120 or C2_020115] fed alone and the same strain separated from co-fed together) was performed using DESeq R package v1.20 [69]. Adjusted *P* value of 0.05 and log_2_ (fold change) of 2 were set as the threshold for significantly differential expression [70]. Enrichment analysis of differentially expressed genes was performed by the clusterProfiler v3.4.4 [71].

### Quantitative real-time PCR

Total RNAs were extracted from each sample as described above in the section detailing Transcriptomics analysis using RNAiso Plus (Takara; Dalian, China). Genomic DNA contamination was removed by a genomic DNA elimination reaction using gDNA Eraser in PrimeScript RT reagent kit (Takara) and then cDNA was obtained using the reverse-transcription reaction reagent in the kit. RNA degradation and contamination was monitored on 1% agarose gels. The relative expression of *eutB* (encoding the ethanolamine ammonia-lyase heavy chain) and *eutR* (encoding a HTH-type transcriptional regulator) genes was compared to that of *rpoB* using the 2^-ΔΔCt^ algorithm [72] using quantitative real-time PCR (qRT-PCR) with primers shown in Supplementary Table S6. The above experiments were repeated nine times with three biologic replicates and three technical replicates for each biologic replicate.

### Ethanolamine utilization assay

Utilization of ethanolamine as a sole nitrogen source of strain C1_020120 and C2_020115 was determined by bacterial growth with addition of 5 mM ethanolamine hydrochloride (Macklin; Shanghai, China) and 0.1% glucose to the modified M9 (mM9) minimal medium. The mM9 medium was a minimal medium containing 48 mM Na_2_HPO_4_, 22 mM KH_2_PO_4_, and 8.5 mM NaCl supplemented with 1 mM MgSO_4_, 0.1 mM CaCl_2_, 150 nM vitamin B12, 5 mg/L vitamin B1, and 2 ml/L trace metals (0.1 μM ZnSO_4_, 0.045 μM FeSO_4_, 0.2 μM Na_2_MoO_4_, 2 μM MnSO_4_, 0.1 μM CuSO_4_, 3 μM CoCl_2_, and 0.1 μM NiSO_4_). Utilization of ethanolamine as a sole carbon source was investigated using the mM9 medium supplemented with 5 mM ethanolamine and 20 mM NH_4_Cl [73]. A single colony of each strain was incubated in 5 ml LB medium overnight at 37 °C. Pellets harvested by centrifugation from 2 ml culture medium at 10,000 r/min for 2 min were suspended with 1 ml mM9 medium and measured to the 0.5 McFarland standard. A 100 μl 1:10^5^ dilution was added into each 15 ml growth condition described above in which ethanolamine was utilized as a nitrogen or a carbon source and incubated at 37 °C. OD_600 nm_ was measured every 2 h. The above experiments were performed in triplicates.

### Screening the presence and the orientation of the 160-kb large region in *K. pneumoniae* complete genomes

The 160-kb region in reversion of strain C1_020120 was retrieved and was blasted against all *K. pneumoniae* genomes labelled with complete or chromosome (accessed on 2021-10-28) available in GenBank. Assemblies with anomalous or failed taxonomy check were excluded in the downstream analysis. A minimum threshold of 90% was used for both identity and coverage to determine the presence of target sequences. The orientation of the genome and target regions were determined with the help of flanking regions and housekeeping genes from the multi-locus sequence typing (MLST) scheme of *K. pneumoniae* (https://pubmlst.org/).

## Supplementary information

Supplementary material

Dataset S1

Dataset S2

Dataset S3

Dataset S4

Dataset S5

Dataset S6

## Data Availability

All data generated or analyzed during this study are included in this article and its supplementary files. Figures 2, 3, and 6 are associated with raw data, which are available as Supplementary datasets. **Nucleotide sequence accession no**. Draft whole-genome sequences of the strains have been deposited into GenBank under accession numbers listed in Dataset S1 in the Supplementary file. The complete sequences of the chromosome and plasmids of strain C2_020115, C1_020120, and 020130 have been deposited in GenBank with the accession numbers CP043357 to CP043361, CP043353 to CP043356, and CP043362 to CP043366, respectively.
