# Supplementary material for: Arm race among closely-related carbapenem-resistant Klebsiella pneumoniae clones
Source: ISME Commun. 2022 Aug 22;2:76. doi: 10.1038/s43705-022-00163-y (PMC9723571; doi:10.1038/s43705-022-00163-y)
Supplement: Supplementary file 1 — Supplementary material [file 43705_2022_163_MOESM1_ESM.docx]

**Supplementary files**

**List**

**Text S1.** OmpK35 and Omp36 porin deficiency of two strains without carbapenemase genes.

**Text S2.** Clone 1 has 12 unique SNPs and a truncated *ulaB* and lacks a 10.5-kb region, but these features are unlikely to explain its successful spread.

**Dataset S1.** CRKP isolates recovered in the ICU during the study period (between March 27 and August 31, 2017).

**Dataset S2.** SNPs between ST11 CRKP isolates.

**Dataset S3.** ST11 KL64 CRKP clinical isolates from the hospital before March 27, 2017.

**Dataset S4.**  Genes upregulated.

**Dataset S5.** Differential expression in ethanolamine, 1,2-propanediol, and cobalamin pathways.

**Dataset S6.** The orientation of the 160-kb region in *K. pneumoniae* complete genomes.

**Fig. S1.** The interruption of *ulaB* in clone 1 isolates by the insertion of IS*Kpn26*.

**Fig. S2.** The 10.5-kb region absent from clone 1.

**Fig. S3.** *In vitro* survival.

**Fig. S4.** qRT-PCR for *eutB* and *eutR* genes between strain 020120 and 020115 in separated culture in broth and after passage in murine gut.

**Table S1.** Allele types of the six STs seen in this study.

**Table S2.** The complete genome and antimicrobial resistance genes of strains 020120, 020115, and 020130.

**Table S3.** SNPs unique to clone 1.

**Table S4.** Genes in the 10.5-kb region absent from clone 1.

**Table S5.** Functional homologs of the products encoded by genes absent from isolates of clone 1.

**Table S6**. Primers used for PCR.

**Text S1. OmpK35 and Omp36 porin deficiency of two strains without carbapenemase genes.**

In strain 015642, OmpK35 was inserted by an IS*Ecp1* at nucleotide position 82 and there was a missense mutant in OmpK36 which resulted in a premature stop codon (nucleotide position 586 C to T [C586T] resulting in amino acid position 196 from Gln to a stop codon [Gln196*]).

In strain 015643, OmpK35 was truncated due to a missense mutant (C520T resulting in Gln174*). There were multiple mutations of OmpK36 in 015643 including 5 missense variations and a premature truncation (C937CT resulting in Gln313*).

**Text S2. Clone 1 has 12 unique SNPs and a truncated *ulaB* and lacks a 10.5-kb region, but these features are unlikely to explain its successful spread**

To understand the genetic basis of the successful spread of clone 1, we compared genome sequences of all isolates of clone 1 with those of all other clones seen in the ICU to identify SNPs and genes that were specific for clone 1.

Compared to those of other clones in the ICU, isolates of clone 1 had 13 unique SNPs including 11 in protein coding sequences (CDS) and 2 in a spacer region (Supplementary Table S3). The clone-specific SNP in the spacer region was found between downstream of *mutS* (encoding DNA mismatch repair protein MutS) and downstream of a gene encoding Hok family toxin of an antitoxin-toxin system and was not present in any promoter regions. Among the 11 SNPs in CDS, six were missense mutations leading to amino acid substitutions and five were synonymous. Of note, one of the missense mutations occurred in *rcsC*, which encodes a sensor kinase as part of the Rcs phosphorelay. The Rcs phosphorelay is conserved throughout the *Enterobacteriaceae* as an important signaling pathway and has also been found to actively participate in biofilm formation^1^ and environmental survival^2^. Mutations of *rcsC* have been associated with decreased biofilm formation^3,4^ and also influence the survival in environment^5^. In addition, there were three consecutive additional nucleotides inserted in *igaA* (intracellular growth attenuation), which lead to one more aa at position 662 of the 709-aa IgaA protein in isolates of clone 1 than that in other clones. *igaA* has been found to negatively regulate the Rcs phosphorelay^6-8^. The alteration of IgaA may lead to further repressed expression of the Rcs phosphorelay and therefore contributes to the decreased biofilm formation in clone 1 (see below).

All clone 1 isolates had an *ulaB* gene interrupted by the insertion of IS*Kpn26* (Supplementary Fig. S1), while all isolates of other clones had an intact *ulaB*. *ulaB* (previously known as *sgaB*) is part of the *ulaABC* regulon and encodes an IIB-like enzyme, which is required for the uptake and utilization of L-ascorbate (vitamin C) under both anaerobic^9^ and aerobic conditions^10^. The interrupted *ulaB* suggests that clone 1 isolates were unable to utilize L-ascorbate. Although L-ascorbate can serve as a carbon source, it is well known that L-ascorbate can also act as an iron-reducing agent and may lead to toxicity effects against host bacterial cells by triggering the Fenton reaction to cause damaging oxidative stress^11,12^. It remains to be determined whether the inability of utilization of L-ascorbate is a defensive mechanism of *K. pneumoniae* to adapt the gut of ICU patients.

Compared to isolates of other clones, a 10.6-kb region was missed from all clone 1 isolates (Fig. 4). An insertion sequence IS*Kpn26* was present in the missing region and truncated a *fimD* gene in clone 1 isolates, while there are multiple copies of IS*Kpn26* present in the corresponding region in isolates of clone 2 (Fig. 4). Therefore, the missing of this region in clone 1 is likely due to homologous recombination between two or more copies of insertion sequence IS*Kpn26*. This region contains part of *fimD* and three intact genes involving in the synthesis of type 1 fimbriae. However, there is another intact *fim* gene cluster encoding the synthesis of type 1 fimbriae in the chromosome of all clone 1 isolates. In addition, a previously study has found that type 1 fimbriae did not influence the ability of *K. pneumoniae* to colonize the gut^13^. This 10.6-kb region also contains six genes encoding proteins for metabolisms or amino acid synthesis (Supplementary Table S4), but clone 1 isolates also had functional homologs of the products encoded by four of the six genes (Supplementary Table S5).

**Fig. S1. The truncation of *ulaB* in clone 1 isolates by the insertion of IS*Kpn26*.**

**Fig. S2. The 10.5-kb region absent from clone 1.** Strains of clone 1 (ST11 KL64) lack a 10.5-kb region compared with those of other clones of ST11, which are shown as non-clone 1 here. The 10.5-kb region is enlarged to show the genes in it and the corresponding region of strain 020130 of clone 2 is shown here to demonstrate the gene names with gene function listed in Table S5. There are multiple copies of IS*Kpn26* at the adjacent of this 10.50kb region.

**Fig. S3. *In vitro* survival.** *In vitro* survival assays mimicking the ICU environment were performed as described previously^14,15^. Survival days were determined using the last day of bacterial growth in at least two of the three replicates. *E. coli* ATCC 25922 survived only one to two days, while strain 020120 (clone 1) and 020115 (clone 2) survived 7 and 11 days, respectively.

**Fig. S4.** qRT-PCR for *eutB* and *eutR* genes between strain 020120 and 020115 in separated culture in broth and after passage in murine gut (feces). There were no significant differences in the expression of *eutB* and *eutR* between 020120 and 020115 in broth culture (*P* = 0.372 and *P* = 0.368) nor after passage alone in murine gut (*P* = 0.492 and *P* = 0.481).

Table S1. Allele types of the six STs seen in this study

| ST | *gapA* | *infB* | *mdh* | *pgi* | *phoE* | *rpoB* | *tonB* |
| --- | --- | --- | --- | --- | --- | --- | --- |
| 11 | 3 | 3 | 1 | 1 | 1 | 1 | 4 |
| 37 | 2 | 9 | 2 | 1 | 13 | 1 | 16 |
| 45 | 2 | 1 | 1 | 6 | 7 | 1 | 12 |
| 292 | 2 | 1 | 2 | 1 | 1 | 1 | 4 |
| 661 | 4 | 3 | 1 | 36 | 9 | 10 | 14 |
| 1640 | 3 | 3 | 1 | 1 | 1 | 9 | 4 |

Table S2. The complete genome and antimicrobial resistance genes of strains 020120, 020115, and 020130.

|  | Accession no. | Size, bp | Replicon type | Genes mediating resistance to | | | | | | |
| --- | --- | --- | --- | --- | --- | --- | --- | --- | --- | --- |
|  |  |  |  | β-lactam | Aminoglycoside | Macrolide | Rifampin | Sulfonamide | Tetracycline | Trimethoprim |
| 020120 (clone 1) |  |  |  |  |  |  |  |  |  |  |
| Chromosome | CP043357 | 5,462,355 |  | *bla*_SHV-11_ | *aadA2b* |  |  |  |  |  |
| pKPC2_020120 | CP043358 | 154,719 | IncFII_pHN7A8_, IncR | *bla*_TEM-1_, *bla*_KPC-2_, *bla*_CTX-M-65_ | *rmtB* |  |  |  |  |  |
| p1_020120 | CP043359 | 205,652 | IncFIB_K_, IncFII_K_ |  | *aadA2, aph(3')-Ia* | *mph(A)* |  | *sul1* |  | *dfrA12* |
| p2_020120 | CP043360 | 10,060 | ColRNAI |  |  |  |  |  |  |  |
| p3_020120 | CP043361 | 5,596 | ND |  |  |  |  |  |  |  |
| 020115 (clone 2) |  |  |  |  |  |  |  |  |  |  |
| Chromosome | CP043353 | 5,446,703 |  | *bla*_SHV-11_ | *aadA2b* |  |  |  |  |  |
| pKPC2_020115 | CP043354 | 99,284 | IncFII_pHN7A8_, IncN | *bla*_TEM-1_, *bla*_KPC-2_, *bla*_CTX-M-65_ | *aac(6')-Ib-cr* |  |  |  | *tet(A)* |  |
| p1_020115 | CP043355 | 78,836 | IncR |  | *aadA16* |  | *arr-3* | *sul1* |  | *dfrA27* |
| p2_020115 | CP043356 | 5,596 | ND |  |  |  |  |  |  |  |
| 020130 (the closest strain of clone 1) | | | | | | | | | | |
| Chromosome | CP046962 | 5,469,023 |  | *bla*_SHV-11_ | *aadA2* |  |  | *sul1* |  |  |
| pKPC2_020130 | CP046963 | 152,833 | IncFII_pHN7A8_, IncR | *bla*_TEM-1_, *bla*_KPC-2_, *bla*_CTX-M-65_ | *rmtB1* |  |  |  |  |  |
| p1_020130 | CP046964 | 55,161 | ND |  |  |  |  |  |  |  |
| p2_020130 | CP046965 | 10,060 | ColRNAI |  |  |  |  |  |  |  |
| p3_020130 | CP046966 | 5,596 | ND |  |  |  |  |  |  |  |

ND, undetermined.

Table S3. SNPs unique to clone 1.

| Position | Type | Ref_020115 | Alt_020120 | Nt_Position | Aa_Position | Effect | Gene | Product |
| --- | --- | --- | --- | --- | --- | --- | --- | --- |
| 385068 | del | ACAG | A | 1986/  2130 | 662/  709 | conservative_inframe_deletion c.1984_1986delCTG p.Leu662del | *igaA* | intracellular growth attenuator family protein |
| 995958 | snp | G | T | 1023/  1389 | 341/  462 | synonymous_variant c.1023C>A p.Arg341Arg | *pduP* | CoA-acylating propionaldehyde dehydrogenase PduP |
| 1105730 | snp | C | T |  |  | Spacer |  |  |
| 1205030 | snp | A | T | 152/  909 | 51/  302 | missense_variant c.152A>T p.Gln51Leu | *lysR* | LysR family transcriptional regulator |
| 1319994 | snp | G | A | 860/  1617 | 287/  538 | missense_variant c.860G>A p.Arg287His | *mltF* | membrane-bound lytic murein transglycosylase MltF |
| 1390885 | snp | G | A | 112/  1950 | 38/  649 | synonymous_variant c.112C>T p.Leu38Leu | - | spore coat protein CotH |
| 1651174 | snp | G | A | 2747/  2841 | 916/  946 | missense_variant c.2747G>A p.Gly916Glu | *rcsC* | two-component system sensor histidine kinase RcsC |
| 1903292 | snp | C | T | 434/  747 | 145/  248 | missense_variant c.434G>A p.Arg145His | *mobC* | molybdopterin-guanine dinucleotide biosynthesis protein MobC |
| 2600369 | snp | C | A | 150/  996 | 50/  331 | synonymous_variant c.150C>A p.Pro50Pro | - | hypothetical protein |
| 3618813 | snp | C | T | 267/  1659 | 89/  552 | synonymous_variant c.267G>A p.Arg89Arg | - | hypothetical protein |
| 3798542 | del | CA | C |  |  | Spacer |  |  |
| 4010037 | snp | G | A | 264/  852 | 88/  283 | synonymous_variant c.264C>T p.Val88Val | - | transporter substrate-binding domain-containing protein |
| 4443847 | snp | T | C | 2408/  2664 | 803/  887 | missense_variant c.2408T>C p.Phe803Ser | *glnD* | bifunctional uridylyltransferase/  uridylyl-removing protein GlnD |

Ref_020115 refers to the nucleotide in strain 020115 (clone 2) and Alt_020120 refers to that in strain 020120 (clone 1). Nt_Position refers to the nucleotide position and Aa_Position to amino acid position. -, unnamed.

Table S4. Genes in the 10.5-kb region absent from clone 1^a^.

| Gene | Location^b^ | Product |  |
| --- | --- | --- | --- |
| *fimD* | 1216319..1218856 | Outer membrane usher protein | WP_004212901.1 |
| *yadV* | 1218886..1219554 | Probable pilin chaperone | WP_002914261.1 |
| *fimA* | 1219596..1220144 | Major type 1 subunit fimbrin (Pilin) | WP_020802021.1 |
| *fimE* | 1220248..1220865 | Putative transcriptional regulator of fimbrial expression | WP_020316947.1 |
| *mltF* | 1221218..1222066 | Cyclohexadienyl dehydratase/putative ABC-type amino acid transport | WP_016831578.1 |
|  | 1222122..1222742 | Putative transport protein | WP_012967387.1 |
| *fabG* | 1222898..1223692 | NAD(P)-dependent short-chain dehydrogenase | YP_004590471.1 |
| *ghrA* | 1223753..1224685 | Glyoxylate/hydroxypyruvate reductase A | WP_014907069.1 |
| *cloR* | 1224691..1225419 | L-fuculose phosphate aldolase | WP_004174769.1 |
| *butD* | 1225420..1226940 | Glutamine ABC transporter ATP-binding protein | WP_012967389.1 |

^a^*fimD* (involving in outer membrane protein export and assembly of type 1 fimbriae) was truncated by IS*Kpn26* and a remnant of *fimD* was present in the region.

^b^Location refers to the chromosome of strain 020115 (GenBank accession no. CP043353).

Table S5. Functional homologs of the products encoded by genes absent from isolates of clone 1.

| Gene absent  from clone 1 | Product | Functional homolog gene  in 020120 chromosome | aa sequence identity | aa sequence coverage |
| --- | --- | --- | --- | --- |
| *cloR* | L-fuculose phosphate aldolase | *otnC* | 27.37% | 73.97% |
| *fabG* | NAD(P)-dependent short-chain Dehydrogenase | *bdhA* | 30.68% | 100.00% |
| *btuD* | Glutamine transport ATP-binding protein GlnQ | *glnQ_6* | 52.21% | 49.21% |
| *ghrA* | Glyoxylate/hydroxypyruvate reductase A | *ghrA_2* | 42.86% | 88.06% |

Table S6. Primers used for PCR^a^

| primer | Sequence 5’→3’ |
| --- | --- |
| Conventional PCR |  |
| rmtB-F | TATCTTTTCAGCCGAAACTC |
| rmtB-R | TAAGTTCTGTTCCGATGGTC |
| tetA-F | TGGCACTTCAGGAACAAGC |
| tetA-R | CTGACAACGAGCCTCCTTTT |
|  |  |
| qRT-PCR |  |
| KP_rpoB_qPCR_F | TTACACCACTGAGCAGATCC |
| KP_rpoB_qPCR_R | GGCCTTTCTCAACGTACACT |
| KP_eutR_qPCR_F | TTCATCAACGACACACAACC |
| KP_eutR_qPCR_R | GTATATTCGCGGAAGACCTG |
| KP_eutB_qPCR_F | CTCTATAACGACCGGCAAAT |
| KP_eutB_qPCR_R | AGCAGGATCATCAGGTTTTC |

^a^All primers were designed in this study.

**References**

1. Ferrieres L, Clarke DJ. The RcsC sensor kinase is required for normal biofilm formation in *Escherichia coli* K-12 and controls the expression of a regulon in response to growth on a solid surface. *Mol Microbiol* 2003; **50**: 1665-82.

2. Clarke DJ. The Rcs phosphorelay: more than just a two-component pathway. *Future Microbiol* 2010; **5**: 1173-84.

3. Huang YH, Ferrieres L, Clarke DJ. Comparative functional analysis of the RcsC sensor kinase from different Enterobacteriaceae. *FEMS Microbiol Lett* 2009; **293**: 248-54.

4. Nepper JF, Lin YC, Weibel DB. Rcs Phosphorelay Activation in Cardiolipin-Deficient *Escherichia coli* Reduces Biofilm Formation. *J Bacteriol* 2019; **201**.

5. Ophir T, Gutnick DL. A role for exopolysaccharides in the protection of microorganisms from desiccation. *Appl Environ Microbiol* 1994; **60**: 740-5.

6. Wall E, Majdalani N, Gottesman S. The Complex Rcs Regulatory Cascade. *Annu Rev Microbiol* 2018; **72**: 111-39.

7. Cano DA, Martinez-Moya M, Pucciarelli MG, Groisman EA, Casadesus J, Garcia-Del Portillo F. *Salmonella enterica* serovar Typhimurium response involved in attenuation of pathogen intracellular proliferation. *Infect Immun* 2001; **69**: 6463-74.

8. Garcia-Calderon CB, Casadesus J, Ramos-Morales F. Regulation of *igaA* and the Rcs system by the MviA response regulator in *Salmonella enterica*. *J Bacteriol* 2009; **191**: 2743-52.

9. Zhang Z, Aboulwafa M, Smith MH, Saier MH, Jr. The ascorbate transporter of *Escherichia coli*. *J Bacteriol* 2003; **185**: 2243-50.

10. Campos E, de la Riva L, Garces F, et al. The *yiaKLX1X2PQRS* and *ulaABCDEFG* gene systems are required for the aerobic utilization of L-ascorbate in *Klebsiella pneumoniae* strain 13882 with L-ascorbate-6-phosphate as the inducer. *J Bacteriol* 2008; **190**: 6615-24.

11. Imlay JA. Pathways of oxidative damage. *Annu Rev Microbiol* 2003; **57**: 395-418.

12. Meneghini R. Iron homeostasis, oxidative stress, and DNA damage. *Free Radic Biol Med* 1997; **23**: 783-92.

13. Struve C, Bojer M, Krogfelt KA. Characterization of *Klebsiella pneumoniae* type 1 fimbriae by detection of phase variation during colonization and infection and impact on virulence. *Infect Immun* 2008; **76**: 4055-65.

14. Havill NL, Boyce JM, Otter JA. Extended survival of carbapenem-resistant *Enterobacteriaceae* on dry surfaces. *Infect Control Hosp Epidemiol* 2014; **35**: 445-7.

15. Wendt C, Dietze B, Dietz E, Ruden H. Survival of *Acinetobacter baumannii* on dry surfaces. *J Clin Microbiol* 1997; **35**: 1394-7.
